# Psychological safety and perceived risk are associated with emergency nurses’ intention to use AI-augmented triage systems

**DOI:** 10.1038/s41598-026-56833-7

**Published:** 2026-06-10

**Authors:** Yuan Jiang, Minghao Kong, Taotao Feng, Shijie Wu

**Affiliations:** 1 School of Medicine, Shanghai Tenth People’s Hospital, Tongji University, Shanghai, China; 2 Tongji University School of Medicine, Shanghai, China

**Keywords:** Artificial intelligence, Emergency nursing, Psychological safety, Health informatics, Technology adoption, Perceived risk, Health care, Psychology, Psychology

## Abstract

Emergency department overcrowding places sustained pressure on triage workflows and patient prioritization. Artificial intelligence (AI)-augmented triage systems have been introduced to support emergency decision-making, but frontline adoption may depend on both technology-related perceptions and human-organizational conditions. This study examined factors associated with emergency nurses’ attitudes and intention to use AI-augmented triage systems, with particular attention to psychological safety and perceived risk. A multi-hospital cross-sectional survey was conducted among 162 frontline triage nurses across nine pilot hospitals in Shanghai between June and August 2025. All participants had at least six months of emergency triage experience and at least three months of actual experience using the AI-augmented triage system. Partial least squares structural equation modelling was used to assess the measurement and structural models. The model explained 57.2% of the variance in attitude and 41.0% of the variance in intention to use. Task-technology fit (β = 0.483, 95% CI [0.387, 0.574]), perceived explainability (β = 0.385, 95% CI [0.280, 0.484]), and psychological safety (β = 0.401, 95% CI [0.294, 0.512]) were positively associated with attitude. Attitude was positively associated with intention to use (β = 0.629, 95% CI [0.526, 0.710]). Perceived risk showed a small negative moderating effect on the association between attitude and intention to use (β = − 0.139, *p* = 0.039, f^2^ = 0.029), although the 95% confidence interval included zero. Common method bias was assessed using procedural and statistical checks; however, same-source bias could not be fully ruled out. Emergency nurses’ intention to use AI-augmented triage systems was associated with both technology-related perceptions and human-organizational conditions. Psychological safety was positively associated with attitudes toward AI use, while perceived risk may modestly weaken the translation of favourable attitudes into intention to use. Given the cross-sectional and self-reported design, the findings should be interpreted as associations rather than causal evidence. Implementation strategies should address task fit, explainability, accountability boundaries, and psychologically safe human–AI collaboration.

## Introduction

Emergency department (ED) overcrowding is not merely a logistical issue but a persistent clinical workflow and patient safety challenge^1,2^. It compromises timely care, increases decision-making pressure on triage nurses, and contributes to workforce strain and burnout^3,4^. To address these operational challenges, hospitals are increasingly implementing artificial intelligence (AI)-augmented triage systems to support risk stratification, patient prioritization, and clinical workflow optimization in emergency care^5^.

AI-augmented triage differs from conventional digital tools because it introduces algorithmic recommendations into time-sensitive clinical judgement. In emergency triage, nurses must rapidly assess patient urgency while dealing with incomplete information, high workload, and accountability for prioritization decisions. Although AI systems may support consistency and efficiency, their use also introduces new forms of uncertainty. Nurses may need to interpret recommendations generated by complex models, decide whether to rely on or override algorithmic outputs, and remain accountable for the final triage decision. Therefore, adoption of AI-augmented triage depends not only on technical performance and infrastructure, but also on how frontline nurses perceive explainability, task fit, psychological safety, and risk in human–AI collaboration.

Existing technology acceptance theories, including the technology acceptance model, the theory of planned behavior, and task-technology fit theory, provide useful foundations for understanding users’ attitudes and behavioural intentions toward health technologies^6–9^. These frameworks suggest that users are more likely to accept a system when it is perceived as useful, compatible with task requirements, and supportive of workflow needs. However, AI-augmented triage occurs in a high-stakes clinical setting in which adoption is shaped by more than general perceptions of usefulness or technical readiness. Unlike many conventional clinical decision support tools, AI-augmented triage may involve algorithmic opacity, human–AI disagreement, and distributed accountability among nurses, clinicians, administrators, and technology providers^10–12^. These features make emergency AI adoption not only a technology acceptance issue, but also an implementation, accountability, and human–AI collaboration issue.

Psychological safety is particularly relevant in this context. Edmondson^13^ defines psychological safety as a shared belief that a team is safe for interpersonal risk-taking. In the present study, psychological safety is applied to the AI-augmented triage context and refers to nurses’ perceived ability to use, question, override, or report concerns about AI-generated triage recommendations without fear of blame, punishment, or negative professional evaluation. Thus, psychological safety is conceptualized as an individual perception of the local implementation climate rather than as a hospital-level organizational culture variable. In emergency care, such perceptions may influence whether nurses feel able to engage critically with AI outputs or whether they avoid using the system defensively because of fear of accountability. This perspective is consistent with implementation research suggesting that technology adoption in healthcare depends on both technical functionality and the social conditions under which users are expected to incorporate new tools into practice^14–16^.

Perceived risk is another important factor in AI-augmented triage adoption. In technology adoption research, perceived risk refers to users’ concerns about potential negative consequences of system use^17,18^. In emergency nursing, such concerns may include fear of clinical errors, legal ambiguity, professional liability, and uncertainty about responsibility when AI-supported recommendations are later judged to be inappropriate. In addition, ethical and societal concerns surrounding healthcare AI, including fairness, accountability, privacy, and unequal performance across patient subgroups, may further shape clinicians’ perceptions of risk^19^. Therefore, perceived risk may not only be a direct concern about system use, but may also condition whether favourable attitudes toward AI-augmented triage translate into intention to use the system.

This study examines emergency nurses’ adoption of AI-augmented triage systems in nine pilot hospitals in Shanghai. It contributes to the literature in three ways. First, it contextualizes established technology acceptance constructs within a high-stakes emergency triage setting, where algorithmic recommendations are embedded in rapid clinical judgement and professional accountability. Second, it conceptualizes psychological safety as a human and organizational condition associated with nurses’ attitudes toward AI-supported triage, rather than treating adoption as a purely technical or usability issue. Third, it examines perceived risk as a potential boundary condition in the relationship between attitude and intention to use. By doing so, this study aims to clarify how technology-related perceptions and human-risk perceptions are associated with emergency nurses’ intention to use AI-augmented triage systems.

## Literature review and hypotheses

### Attitude and intention to use

Attitude has long been regarded as a proximal antecedent of behavioural intention in technology adoption research. According to the theory of planned behavior and the technology acceptance model, individuals who evaluate a technology more favourably are more likely to intend to use it in future behaviour^6,7^. In the context of AI-augmented triage, attitude refers to nurses’ overall positive or negative evaluation of using the AI system in their triage work, whereas intention to use refers to their willingness or plan to continue incorporating the system into daily triage practice.

In emergency nursing, a favourable attitude toward AI-augmented triage may develop when nurses perceive the system as beneficial, clinically relevant, and compatible with their professional role. However, because the present study uses a cross-sectional design, the relationship between attitude and intention is conceptualized as an association rather than a causal effect. Therefore, the following hypothesis is proposed:

- H1: Nurses’ attitude toward using AI-augmented triage is positively associated with their intention to use the system.

### Task-technology fit and attitude

Task-technology fit theory suggests that users are more likely to evaluate a technology favourably when its functions match the requirements of the tasks they need to perform^8^. Emergency triage is characterized by time pressure, incomplete information, multitasking, and the need for rapid prioritization. For AI-augmented triage to be viewed positively by nurses, the system must fit these task demands by supporting efficient assessment, prioritization, and workflow continuity.

In this study, task-technology fit refers to the extent to which nurses perceive the AI-augmented triage system as compatible with their daily triage workflow and clinical task requirements. When nurses perceive that the system supports rather than disrupts triage work, they are more likely to form a favourable attitude toward its use. Therefore, the following hypothesis is proposed:

- H2: Task-technology fit is positively associated with nurses’ attitude toward using AI-augmented triage.

### Perceived explainability and attitude

Explainability is a central issue in healthcare AI because clinicians are often expected to make decisions based on outputs generated by complex or opaque algorithms. In clinical decision support, lack of explainability may reduce trust, increase uncertainty, and make it difficult for clinicians to justify or challenge system recommendations^10,11^. Conversely, when AI systems provide understandable reasons for their recommendations, clinicians may be more likely to evaluate them as trustworthy and clinically useful^20,21^.

In the context of emergency triage, perceived explainability refers to nurses’ perception that the AI system provides clear, understandable, and transparent reasons for its recommended triage category. Explainability is especially important because triage nurses remain responsible for interpreting patient urgency and may need to justify why they accept or override AI-generated recommendations. Therefore, perceived explainability may be positively associated with nurses’ attitude toward using AI-augmented triage. Accordingly, the following hypothesis is proposed:

- H3: Perceived explainability is positively associated with nurses’ attitude toward using AI-augmented triage.

### Psychological safety and attitude

Psychological safety refers to a shared belief that a team or work environment is safe for interpersonal risk-taking^13^. In healthcare settings, psychological safety is closely related to learning behaviour, error reporting, speaking up, and the ability to discuss uncertainty without fear of blame or punishment^14^. These features are particularly relevant when new technologies are introduced into clinical workflows, because users may encounter uncertainty, unexpected errors, or situations in which system outputs conflict with professional judgement.

In this study, psychological safety is applied specifically to the AI-augmented triage context. It refers to nurses’ perceived ability to use, question, override, or report concerns about AI-generated triage recommendations without fear of blame, punishment, or negative professional evaluation. This conceptualization differs from general organizational culture because it focuses on nurses’ individual perception of the local implementation climate surrounding AI use. When nurses perceive that their department supports learning from AI-related uncertainty and does not blame individuals for good-faith engagement with new technology, they may be more willing to evaluate the AI system positively.

Recent nursing and healthcare AI literature also suggests that frontline clinicians’ engagement with AI depends not only on technical literacy, but also on professional confidence, organizational support, and the ability to navigate uncertainty during implementation^16,22^. Therefore, the following hypothesis is proposed:

- H4: Psychological safety is positively associated with nurses’ attitude toward using AI-augmented triage.

### The moderating role of perceived risk

Perceived risk refers to users’ concerns about potential negative consequences associated with technology use^18^. In healthcare contexts, perceived risk may include concerns about system failure, data security, clinical error, legal ambiguity, and professional accountability^17^. In emergency triage, these concerns may be particularly salient because triage decisions influence patient prioritization, waiting time, and escalation of care. Even when nurses hold favourable attitudes toward AI-augmented triage, high perceived risk may make them less willing to translate that attitude into intention to use the system.

Perceived risk is also relevant because AI-supported triage involves human–AI collaboration rather than simple tool use. Nurses may face situations in which their clinical judgement differs from AI-generated recommendations. In such cases, perceived risk may arise from uncertainty about who is accountable for the final decision, how errors will be evaluated, and whether the system performs equitably across diverse patient groups. Ethical discussions of healthcare AI highlight that explainability, fairness, and accountability are central to clinicians’ trust and perceived legitimacy of AI-supported decisions^19^. Therefore, perceived risk may function as a boundary condition that weakens the association between favourable attitudes and intention to use AI-augmented triage.

Accordingly, the following hypothesis is proposed:

- H5: Perceived risk negatively moderates the association between nurses’ attitude toward AI-augmented triage and their intention to use the system.

## Summary of the conceptual framework

**Fig. 1:**
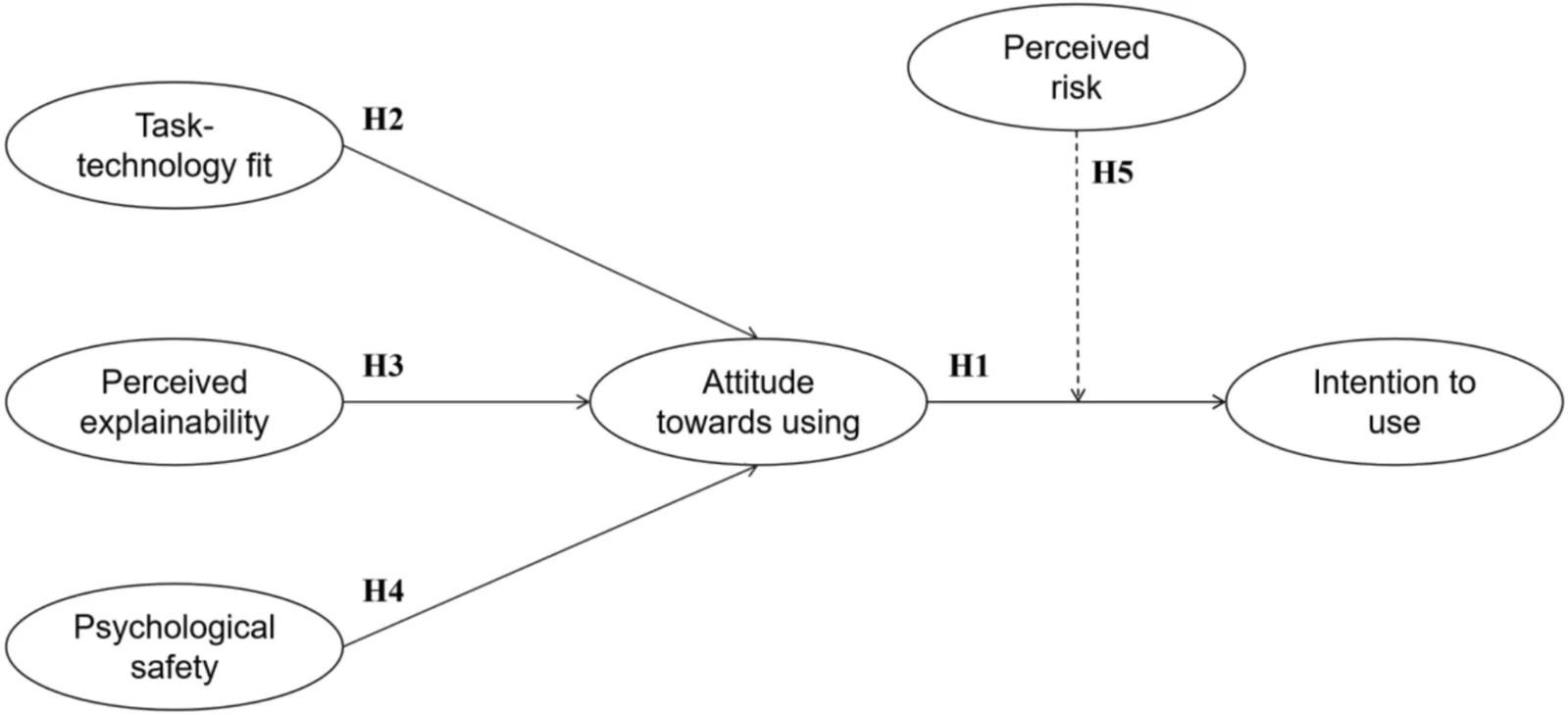
Proposed conceptual framework for emergency nurses’ adoption of AI-augmented triage systems. The model hypothesizes that attitude toward using AI-augmented triage is positively associated with intention to use. Task-technology fit, perceived explainability, and psychological safety are proposed as positive correlates of attitude. Perceived risk is proposed to negatively moderate the association between attitude and intention to use. *AT* attitude; *IU* intention to use; *TTF* task-technology fit; *PE* perceived explainability; *PS* psychological safety; *PR* perceived risk.

Together, these hypotheses address the study aim by examining technology-related, human-organizational, and risk-related factors associated with emergency nurses’ adoption of AI-augmented triage systems. H1 examines the proximal association between attitude and intention to use. H2 and H3 examine technology-related perceptions, namely task-technology fit and perceived explainability, as correlates of attitude. H4 examines psychological safety as a human-organizational condition associated with attitude. H5 examines perceived risk as a potential boundary condition in the translation of favourable attitudes into intention to use. The proposed conceptual framework is shown in Fig. 1.

## Method

### Study design and setting

This study used a multi-hospital cross-sectional survey design to examine factors associated with emergency nurses’ intention to use AI-augmented triage systems. Data were collected between June 2025 and August 2025 from nine pilot emergency departments in Shanghai, China, where the same AI-augmented triage system had been implemented.

The participating hospitals were selected because they were among the early pilot sites using the AI-augmented triage system in emergency care. This setting allowed the study to examine nurses’ adoption perceptions in hospitals with comparable digital infrastructure and similar system implementation conditions. At the time of data collection, all included participants had used the AI-augmented triage system for at least three months, ensuring that their responses reflected actual system experience rather than initial unfamiliarity with the technology.

### AI-augmented triage system

The AI-augmented triage system was designed to support emergency triage nurses in patient prioritization and risk stratification. The system was integrated with the hospital information system (HIS) and could automatically extract patient vital signs. It also used natural language processing to analyse patients’ chief complaints and, together with machine learning algorithms, generated a recommended triage category from Level I to Level IV based on the Emergency Severity Index.

The system functioned as a clinical decision-support tool rather than an autonomous decision-maker. It generated a recommended triage level and supporting information for nurses’ consideration, but the final triage decision remained the responsibility of the triage nurse according to hospital protocols. Nurses could accept, question, or override the AI-generated recommendation based on direct patient observation, clinical judgement, and institutional triage rules. Physicians were not routinely responsible for the initial triage decision in this workflow, although they became involved in subsequent diagnosis and treatment after triage. During the study period, the system did not autonomously update its decision rules in real time at the point of care.

### Participants and recruitment

The target population consisted of registered nurses working at emergency triage desks in the nine participating pilot hospitals. Eligible participants were required to meet the following criteria: (1) holding a valid registered nurse qualification and currently working at an emergency triage desk; (2) having at least six months of emergency triage experience, to ensure basic familiarity with triage workflow and decision-making requirements; and (3) having used the AI-augmented triage system for at least three months, to ensure that their perceptions of risk, explainability, attitude, and intention were based on actual experience rather than initial unfamiliarity with the technology. Nurses who were not assigned to triage duties or who had used the AI-augmented triage system for less than three months were excluded.

All 196 eligible triage nurses across the nine pilot hospitals were invited to complete the questionnaire through the hospital intranet. A total of 171 questionnaires were returned. After excluding incomplete or invalid responses, 162 valid questionnaires were retained for analysis, yielding an effective response rate of 82.7%.

### Ethical considerations

This study was approved by the Ethics Committee of Shanghai Tenth People’s Hospital, School of Medicine, Tongji University (Approval No. 24KT115). All methods were performed in accordance with the relevant guidelines and regulations. All participants provided informed consent before completing the questionnaire. Participation was voluntary and anonymous, and respondents were informed that they could withdraw at any time without any consequences.

### Instrument development and content validation

The questionnaire was developed based on established scales and adapted to the context of AI-augmented emergency triage. Items for psychological safety were adapted from Edmondson^13^, focusing on nurses’ perceived ability to use, question, or report concerns about the AI system without fear of blame. Items for perceived risk were adapted from Featherman and Pavlou^18^, with wording modified to reflect clinical error, legal ambiguity, and professional accountability in AI-assisted triage. Items for task-technology fit, perceived explainability, attitude, and intention to use were adapted from prior technology acceptance and explainable AI studies^8,23^. All items were measured on a seven-point Likert scale ranging from 1 = strongly disagree to 7 = strongly agree.

Because the questionnaire was administered in Chinese, the standard Brislin translation and back-translation procedure was used to ensure cross-cultural semantic equivalence^24^. The original English items were first translated into Chinese by bilingual researchers and then back-translated into English by an independent bilingual translator. Discrepancies between the original and back-translated versions were reviewed and resolved through discussion.

Content validity was assessed by a panel of six experts, including senior emergency physicians, nursing administrators, and medical informatics specialists. The experts evaluated each item for relevance, clarity, and contextual appropriateness for AI-augmented emergency triage. The item-level content validity index values were all above 0.85, indicating acceptable content validity^25^. Before the formal survey, a pilot test was conducted among 18 emergency nurses who were not included in the final sample. The pilot test was used to assess item clarity, readability, and the fluency of the Chinese wording. Minor wording adjustments were made before formal data collection.

### Measures

The final questionnaire included six latent constructs: task-technology fit, perceived explainability, psychological safety, perceived risk, attitude toward using AI-augmented triage, and intention to use. Each construct was measured using three reflective items. Representative items are provided in Appendix A.

Task-technology fit referred to the extent to which nurses perceived that the AI-augmented triage system matched their daily triage workflow and clinical task requirements. Perceived explainability referred to nurses’ perception that the AI system provided understandable reasons for its triage recommendations. Psychological safety referred to nurses’ perceived ability to use, question, or report concerns about AI-generated recommendations without fear of blame or negative professional evaluation. Perceived risk referred to concerns about clinical error, legal ambiguity, and professional accountability when using AI-assisted triage. Attitude referred to nurses’ overall evaluation of using the AI-augmented triage system, while intention to use referred to their willingness to continue using the system in daily triage practice.

### Statistical analysis

Partial least squares structural equation modelling (PLS-SEM) was used to analyse the data. PLS-SEM was selected because the study aimed to examine a prediction-oriented model involving multiple latent constructs and an interaction term in a relatively modest sample. The analysis focused on explained variance, path coefficients, effect sizes, and predictive relevance rather than confirmation of a well-established covariance structure. This approach is appropriate for prediction-oriented technology adoption research and theory extension involving latent constructs^26,27^.

Sample size adequacy was assessed using both a priori power analysis and the conventional 10-times rule. A power analysis using G*Power indicated that detecting a medium effect size (f^2^ = 0.15) with α = 0.05 and statistical power of 0.80 required a minimum sample size of approximately 77 participants^28^. The final sample of 162 exceeded this requirement. The sample also exceeded the 10-times rule, as the maximum number of structural paths directed at an endogenous construct was three.

The analysis proceeded in two stages. First, the measurement model was assessed using indicator loadings, Cronbach’s alpha, composite reliability, average variance extracted, and the heterotrait-monotrait ratio. Second, the structural model was assessed using standardized path coefficients, t values, *p* values, 95% confidence intervals, coefficient of determination values, effect sizes, predictive relevance, and standardized root mean square residual. The significance of path coefficients was assessed using bootstrapping with 5000 resamples in SmartPLS. Standardized coefficients, t values, *p* values, effect sizes, and 95% percentile confidence intervals were examined.

The moderating effect of perceived risk on the association between attitude and intention to use was tested using the interaction term PR × AT in SmartPLS. Because the study was cross-sectional, all structural paths were interpreted as associations rather than causal effects.

Common method bias was addressed using procedural and statistical approaches. Procedurally, participation was voluntary and anonymous, and respondents were assured that there were no right or wrong answers. Statistically, Harman’s single-factor test was conducted, and collinearity diagnostics were examined. However, because all variables were collected from the same respondents at a single time point, common method bias could not be completely ruled out.

Because participants were recruited from nine hospitals, potential hospital-level clustering was examined before structural model testing. Intraclass correlation coefficients were calculated for the six main constructs using hospital as the grouping variable. The ICC values ranged from 0.000 to 0.039, suggesting limited between-hospital variance. Given the small number of hospital clusters, formal multilevel modelling was not conducted because hospital-level parameter estimates would be unstable. Therefore, the main analysis was conducted at the individual respondent level using PLS-SEM, while the potential influence of shared hospital-level implementation context was considered when interpreting the findings.

Non-response bias was assessed by comparing the demographic characteristics of early and late respondents. The first 25% of respondents were compared with the last 25% of respondents using independent-samples t tests and chi-square tests where appropriate. No significant differences were observed in key demographic variables, including age, gender, education level, and years of emergency experience, suggesting that non-response bias was unlikely to be a major concern^29^.

## Results

### Participant characteristics

A total of 162 valid responses were included in the final analysis. The demographic characteristics of participants are shown in Table 1. Most respondents were female, reflecting the gender composition of the emergency nursing workforce in the participating hospitals. Most participants were between 26 and 35 years old and had more than three years of emergency nursing experience, indicating that the sample consisted mainly of nurses with practical experience in emergency triage.

**Table 1:** Demographic characteristics of participants (N = 162).

Characteristic	Category	Frequency (N)	Percentage (%)
Gender	Female	152	93.8
	Male	10	6.2
Age(years)	20–25	12	7.4
	26–30	78	48.1
	31–35	55	34.0
	> 35	17	10.5
Years of emergency experience	< 3 years	22	13.6
	3–5 years	50	30.9
	6–10 years	65	40.1
	> 10 years	25	15.4

Supplementary descriptive checks were conducted to examine whether available demographic variables were associated with the main constructs. Gender differences were examined using independent-samples t tests, while age group and years of emergency experience were examined using one-way ANOVA. As shown in Table 2, no significant differences were observed in task-technology fit, perceived explainability, psychological safety, perceived risk, attitude, or intention to use across gender, age group, or years of emergency experience groups.

**Table 2:** Associations between demographic characteristics and main constructs.

Background variable	Test	TTF p	PE p	PS p	PR p	AT p	IU p
Gender	Independent-samples t test	0.627	0.467	0.919	0.490	0.453	0.776
Age group	One-way ANOVA	0.902	0.663	0.737	0.158	0.883	0.682
Years of emergency experience	One-way ANOVA	0.698	0.385	0.616	0.147	0.830	0.945

*TTF* task-technology fit; *PE* perceived explainability; *PS* psychological safety; *PR* perceived risk; *AT* attitude; *IU* intention to use.

### Measurement model assessment

The measurement model was assessed using indicator loadings, internal consistency reliability, convergent validity, discriminant validity, and collinearity diagnostics. As shown in Table 3, all indicator loadings ranged from 0.803 to 0.964, exceeding the recommended threshold of 0.70^27^. Cronbach’s alpha values ranged from 0.898 to 0.931, and composite reliability values ranged from 0.915 to 0.956, indicating satisfactory internal consistency. Convergent validity was supported because the average variance extracted values ranged from 0.782 to 0.878, exceeding the recommended threshold of 0.50^30^.

**Table 3:** Assessment of reliability and validity.

Construct	VIF	Factor loading	Cronbach’s alpha	CR	AVE
AT			0.92	0.949	0.861
AT1	3.542	0.933			
AT2	3.173	0.924			
AT3	3.34	0.928			
IU			0.91	0.943	0.847
IU1	3.155	0.923			
IU2	3.196	0.932			
IU3	2.831	0.905			
PE			0.931	0.956	0.878
PE1	3.527	0.937			
PE2	3.735	0.931			
PE3	4.218	0.943			
PR			0.898	0.915	0.782
PR1	3.07	0.803			
PR2	2.496	0.964			
PR3	2.879	0.879			
PS			0.917	0.947	0.857
PS1	3.146	0.92			
PS2	3.583	0.932			
PS3	3.11	0.925			
TTF			0.907	0.942	0.843
TTF1	2.961	0.913			
TTF2	2.984	0.921			
TTF3	2.967	0.922			

*AT* attitude; *IU* intention to use; *PE* perceived explainability; *PR* perceived risk; *PS* psychological safety; *TTF *task-technology fit; *CR *composite reliability; *AVE* average variance extracted.

Outer model collinearity was also examined. The outer VIF values ranged from 2.496 to 4.218, below the threshold of 5, suggesting that indicator-level collinearity was within an acceptable range.

### Discriminant validity

Discriminant validity was assessed using the heterotrait-monotrait ratio. As shown in Table 4, all HTMT values were below the conservative threshold of 0.85, supporting discriminant validity among the latent constructs^31^. The highest HTMT value was observed between attitude and intention to use, which was theoretically expected because attitude is the proximal correlate of intention in the proposed model.

**Table 4:** Discriminant validity (HTMT).

	AT	IU	PE	PR	PS	TTF
AT						
IU	0.68					
PE	0.52	0.372				
PR	0.045	0.064	0.086			
PS	0.394	0.334	0.05	0.05		
TTF	0.546	0.373	0.179	0.047	0.131	
PR x AT	0.051	0.102	0.076	0.077	0.009	0.033

*AT* attitude; *IU* intention to use; *PE* perceived explainability; *PR* perceived risk; *PS* psychological safety; *TTF* task-technology fit.

### Common method bias and collinearity assessment

Because all variables were measured using self-reported questionnaires at a single time point, common method bias was assessed using both procedural and statistical approaches. Procedurally, the survey was voluntary and anonymous, and participants were informed that there were no right or wrong answers. Statistically, Harman’s single-factor test was conducted. The first unrotated factor accounted for 33.634% of the total variance, suggesting that no single factor dominated the data^32,33^.

Collinearity diagnostics were also examined. The inner VIF values ranged from 1.004 to 1.045, below the commonly recommended threshold of 3.3, indicating low collinearity among predictor constructs. Nevertheless, because the data were collected from the same respondents at one time point, common method bias cannot be fully ruled out (Table 5).

**Table 5:** Inner model collinearity statistics.

Relationship	VIF
AT → IU	1.004
PE → AT	1.033
PR × AT → IU	1.009
PS → AT	1.018
TTF → AT	1.045

*AT* attitude; *IU* intention to use; *PE* perceived explainability; *PR *perceived risk; PS psychological safety; *TTF* task-technology fit.

### Assessment of hospital-level clustering

Because participants were recruited from nine hospitals, potential hospital-level clustering was examined before structural model testing. As shown in Table 6, the ICC values for the six main constructs ranged from 0.000 to 0.039, suggesting limited between-hospital variance and indicating that most variance occurred at the individual respondent level. Given the small number of hospital clusters, formal multilevel modelling was not conducted because hospital-level parameter estimates would be unstable. These results supported the use of individual-level PLS-SEM as the primary analytic approach, while recognising that hospital-level contextual effects cannot be fully excluded.

**Table 6:** Intraclass correlation coefficients for the main constructs.

Construct	Between-hospital variance	Residual variance	ICC
TTF	0.000	0.867	0.000
PE	0.024	1.114	0.021
PS	0.000	0.990	0.000
PR	0.037	0.905	0.039
AT	0.000	1.134	0.000
IU	0.021	0.787	0.026

*ICC* intraclass correlation coefficient.

### Structural model assessment and hypothesis testing

The structural model was assessed using standardized path coefficients, t values, *p* values, 95% bootstrap confidence intervals, effect sizes, explained variance, predictive relevance, and model fit. The standardized root mean square residual was 0.044, below the recommended threshold of 0.08, indicating acceptable model fit^34^. The model explained 57.2% of the variance in attitude (R^2^ = 0.572) and 41.0% of the variance in intention to use (R^2^ = 0.410). Predictive relevance was supported because Q^2^ values were greater than zero for both attitude (Q^2^ = 0.557, RMSE = 0.676, MAE = 0.551) and intention to use (Q^2^ = 0.295, RMSE = 0.856, MAE = 0.667)^35^.

**Fig. 2:**
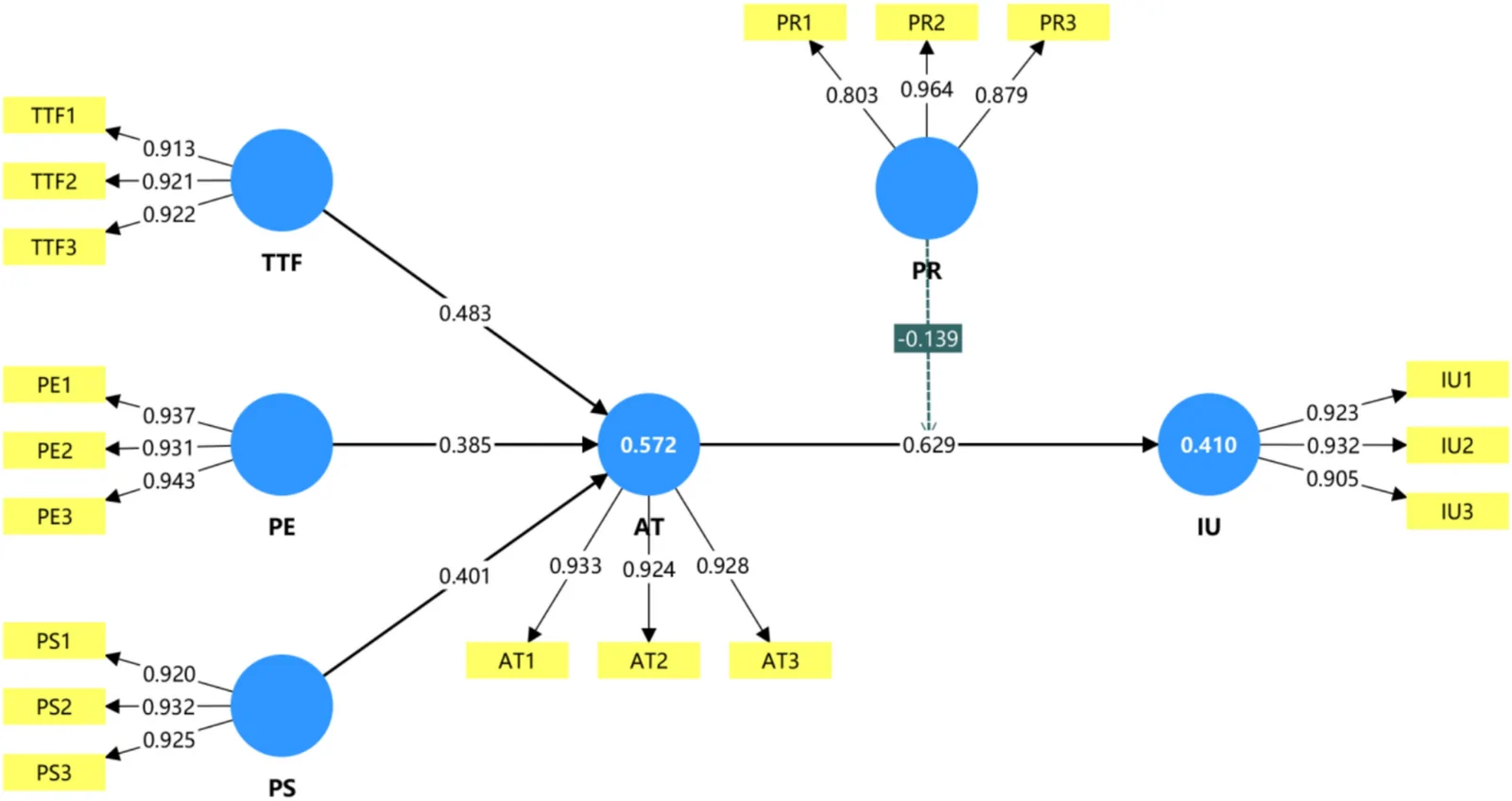
PLS-SEM structural model results. Standardized path coefficients are shown on the arrows, and R^2^ values are shown inside endogenous constructs. The model explained 57.2% of the variance in attitude and 41.0% of the variance in intention to use. Task-technology fit, perceived explainability, and psychological safety were positively associated with attitude. The interaction between perceived risk and attitude showed a small negative association with intention to use. *AT* attitude; *IU* intention to use; *TTF* task-technology fit; *PE* perceived explainability; *PS* psychological safety; *PR* perceived risk.

Hypothesis testing results are presented in Table 7 and visualized in Fig. 2. H1 was supported: attitude toward using AI-augmented triage was positively associated with intention to use the system (β = 0.629, 95% CI [0.526, 0.710], *p* < 0.001). H2 was supported: task-technology fit was positively associated with attitude (β = 0.483, 95% CI [0.387, 0.574], *p* < 0.001). H3 was supported: perceived explainability was positively associated with attitude (β = 0.385, 95% CI [0.280, 0.484], *p* < 0.001). H4 was supported: psychological safety was positively associated with attitude (β = 0.401, 95% CI [0.294, 0.512], *p* < 0.001).

**Table 7:** Structural model results and hypothesis testing.

Hypothesis	Relationship	Β	Mean	SD	t	*p*	95% CI	f^2^	Decision
H1	AT → IU	0.629	0.624	0.047	13.377	< 0.001	[0.526, 0.710]	0.667	Supported
H2	TTF → AT	0.483	0.482	0.048	10.068	< 0.001	[0.387, 0.574]	0.521	Supported
H3	PE → AT	0.385	0.384	0.052	7.358	< 0.001	[0.280, 0.484]	0.335	Supported
H4	PS → AT	0.401	0.404	0.055	7.23	< 0.001	[0.294, 0.512]	0.369	Supported
H5	PR × AT → IU	− 0.139	− 0.137	0.067	2.061	0.039	[− 0.255, 0.021]	0.029	Partially supported

*AT* attitude; *IU* intention to use; *TTF* task-technology fit; *PE* perceived explainability; *PS* psychological safety; *PR* perceived risk. H5 was interpreted cautiously because the interaction effect was small and the 95% confidence interval included zero.

H5 received partial support. The interaction term between perceived risk and attitude was negative and statistically significant based on the bootstrap t-test (β = − 0.139, t = 2.061, *p* = 0.039), suggesting that perceived risk may attenuate the association between attitude and intention to use. However, the 95% bootstrap confidence interval included zero, and the effect size was small (f^2^ = 0.029). Therefore, the moderation effect should be interpreted cautiously as a modest boundary condition rather than a strong moderating mechanism.

### Moderation analysis

**Fig. 3:**
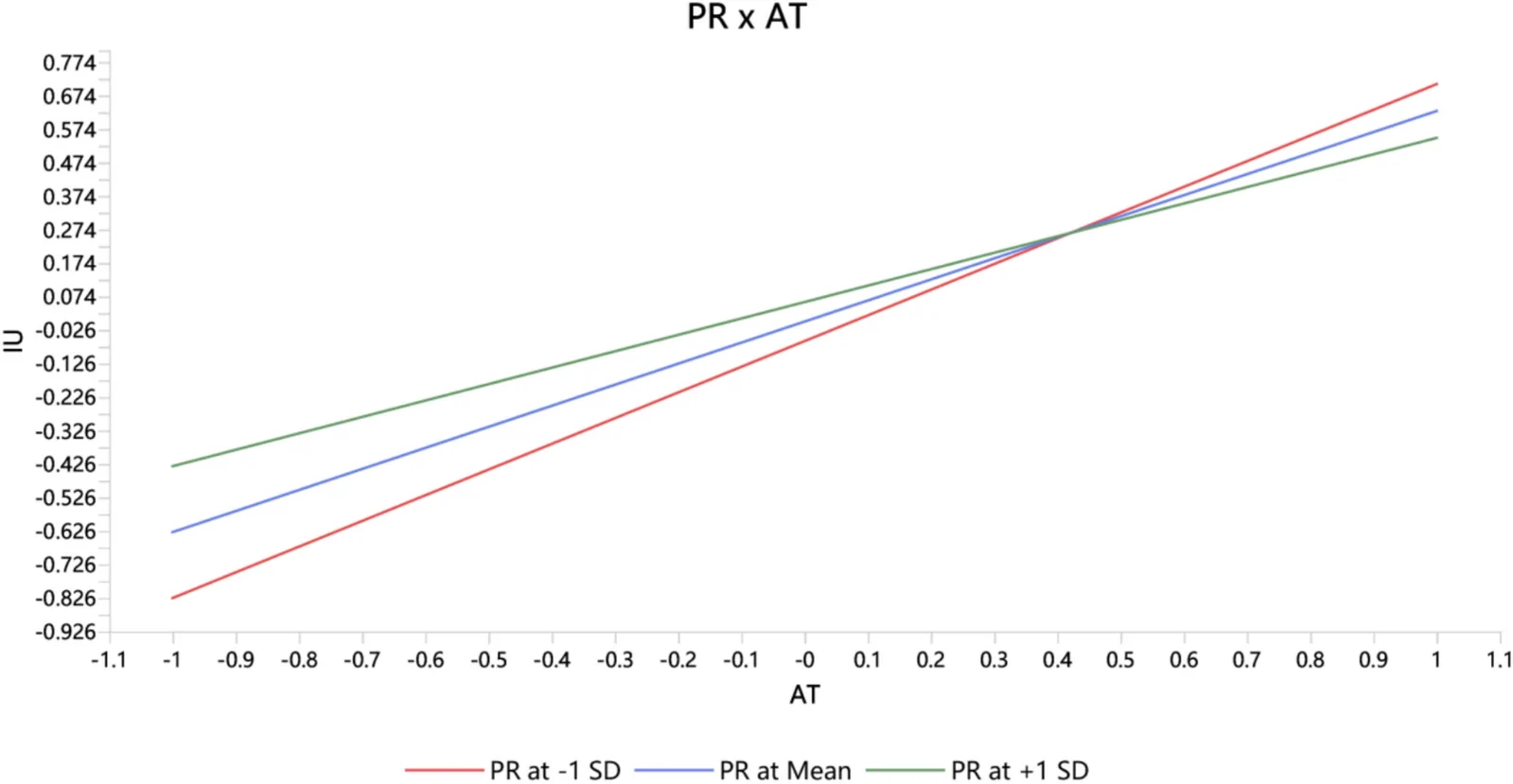
Simple slope analysis of the moderating effect of perceived risk on the association between attitude and intention to use. The figure shows the estimated association between attitude and intention to use at low (− 1 SD), mean, and high (+ 1 SD) levels of perceived risk. The conditional effect decreased from β = 0.767 at low perceived risk to β = 0.629 at mean perceived risk and β = 0.490 at high perceived risk. This pattern suggests that perceived risk may modestly attenuate the translation of favourable attitudes into intention to use. AT = attitude; IU = intention to use; PR = perceived risk.

A simple slope analysis was conducted to interpret the moderating role of perceived risk. As shown in Table 8 and Fig. 3, the conditional effect of attitude on intention to use was strongest at low perceived risk (− 1 SD; β = 0.767, t = 8.982, *p* < 0.001, 95% CI [0.572, 0.913]), remained positive at mean perceived risk (β = 0.629, t = 13.377, *p* < 0.001, 95% CI [0.526, 0.710]), and was weaker at high perceived risk (+ 1 SD; β = 0.490, t = 6.244, *p* < 0.001, 95% CI [0.338, 0.649]). This pattern indicates that the association between attitude and intention to use decreased as perceived risk increased. Therefore, perceived risk may modestly attenuate the extent to which favourable attitudes toward AI-augmented triage are translated into intention to use the system. However, consistent with the small interaction effect size (f^2^ = 0.029) and the confidence interval results reported for H5, this moderation should be interpreted cautiously.

**Table 8:** Conditional effects of attitude on intention to use at different levels of perceived risk.

Perceived risk level	Conditional effect β	t	*P*	95% CI
Low PR (− 1 SD)	0.767	8.982	< 0.001	[0.572, 0.913]
Mean PR	0.629	13.377	< 0.001	[0.526, 0.710]
High PR (+ 1 SD)	0.490	6.244	< 0.001	[0.338, 0.649]

*AT* attitude; *IU* intention to use; *PR* perceived risk; *CI* confidence interval.

## Discussion

### Principal findings

This multi-hospital cross-sectional study examined factors associated with emergency nurses’ intention to use AI-augmented triage systems in nine pilot hospitals in Shanghai. The findings suggest that nurses’ intention to use AI-augmented triage was associated with both technology-related perceptions and human-organizational perceptions. Attitude toward AI-augmented triage showed the strongest association with intention to use, supporting the role of attitude as a proximal correlate of behavioural intention in technology adoption research.

Task-technology fit, perceived explainability, and psychological safety were all positively associated with nurses’ attitudes toward AI-augmented triage. Among these factors, task-technology fit showed the largest path coefficient, suggesting that nurses were more likely to evaluate AI-augmented triage favourably when the system was perceived as compatible with the speed, prioritization demands, and workflow requirements of emergency triage. Perceived explainability was also positively associated with attitude, indicating that understandable AI-generated recommendations may be important when nurses are expected to interpret, validate, or override algorithmic outputs in time-sensitive clinical contexts.

Psychological safety was also strongly associated with nurses’ attitudes toward AI-augmented triage. This finding suggests that adoption perceptions may depend not only on whether the system works technically, but also on whether nurses feel safe to use, question, and report concerns about the system without fear of blame. This is particularly relevant in emergency triage, where nurses remain accountable for prioritization decisions even when AI-generated recommendations are available.

Perceived risk showed a small negative moderating effect on the association between attitude and intention to use. This finding provides cautious support for the idea that risk-related concerns may weaken the translation of favourable attitudes into intention to use AI-augmented triage. However, this moderation effect should not be overstated because the effect size was small and the 95% confidence interval included zero. Therefore, perceived risk is best interpreted as a modest and tentative boundary condition rather than as a strong inhibiting mechanism.

### Theoretical implications

This study contributes to technology adoption research by contextualizing established acceptance constructs within the high-stakes setting of emergency AI-augmented triage. Prior technology acceptance models have emphasized the importance of attitude, perceived usefulness, facilitating conditions, and task-technology fit in shaping behavioural intention^8,9^. The present findings are consistent with this tradition, but they also suggest that AI adoption in emergency care cannot be fully understood as a generic technology acceptance problem.

AI-augmented triage differs from many conventional information systems because it introduces algorithmic recommendations into clinical prioritization decisions that may have immediate patient safety implications. Nurses are not simply deciding whether to use a convenient digital tool; they are engaging with algorithmic outputs in a setting characterized by time pressure, uncertainty, and professional accountability. In this context, explainability and task fit are important because nurses need to understand whether the AI recommendation is relevant, interpretable, and compatible with triage workflow^10,11^.

The findings also support the relevance of psychological safety in AI implementation research. Psychological safety is not treated here as a broad hospital-level culture variable, but as nurses’ individual perception of whether they can engage with AI-generated recommendations without fear of blame or negative professional evaluation. This AI-specific application of psychological safety helps explain why a technically available system may still encounter adoption barriers. If nurses perceive that questioning or overriding AI recommendations could expose them to criticism or liability, they may be less willing to incorporate the system into practice even when they recognize its potential value. This interpretation is consistent with research on psychological safety and technology implementation, which emphasizes the importance of learning-oriented climates when new technologies are introduced into practice^14–16^.

The moderation result adds a further nuance to technology adoption theory. Although attitude was strongly associated with intention to use, perceived risk appeared to modestly weaken this association. This suggests that favourable attitudes may not always translate into intention when clinicians perceive high clinical, legal, or professional uncertainty. However, given the small interaction effect and confidence interval results, this conclusion should be interpreted cautiously. Future research should further examine whether perceived risk functions as a stable boundary condition, whether its role varies across clinical contexts, and whether it has stronger effects in settings with higher AI autonomy or less mature governance structures^36,37^.

### AI-specific implementation and accountability implications

The findings have implications for the implementation of AI-augmented triage systems, but these implications should be interpreted as practice-informed recommendations rather than causal conclusions from the present cross-sectional survey. First, hospitals implementing AI-augmented triage may need to ensure that system functions are closely aligned with the realities of emergency triage work. Technical implementation should focus not only on making AI available, but also on ensuring that recommendations are timely, clinically relevant, and compatible with nurses’ workflow.

Second, explainability should be regarded as more than a technical interface feature. In emergency triage, explainability may support nurses’ ability to evaluate why a particular triage level is recommended, identify potential mismatches between patient presentation and algorithmic output, and justify final decisions. This is especially important because nurses retain responsibility for the final triage decision. Explainability therefore contributes not only to usability, but also to accountability and clinical sense-making in human–AI collaboration^10,11^.

Third, nurse managers and hospital administrators may consider fostering a psychologically safe implementation climate. A just-culture approach may help nurses feel able to report AI-related discrepancies, discuss uncertainty, and learn from implementation problems without fear of individual blame^38,39^. However, such strategies should be evaluated empirically in future implementation studies. The present findings suggest that psychological safety is associated with favourable attitudes, but they do not establish that specific leadership interventions will cause higher AI adoption.

Fourth, perceived risk should be addressed through clear governance rather than informal reassurance alone. Hospitals may consider clarifying responsibility boundaries for AI-assisted triage, including when nurses are expected to accept, question, or override AI-generated recommendations. Human-in-the-loop workflows may be useful when they preserve nurses’ clinical authority and make the validation process explicit. Similarly, just-culture or safe-harbor mechanisms may be worth evaluating for situations in which nurses follow institutional protocols in good faith but AI recommendations later prove inaccurate. These approaches should be framed as future implementation and governance strategies rather than direct conclusions established by the current survey^40,41^.

### Ethical, fairness, and human: AI collaboration considerations

The adoption of AI-augmented triage should also be understood within broader ethical and societal debates about healthcare AI. Perceived risk may not only reflect fear of personal liability or technical error, but also awareness that AI systems may perform unequally across patient subgroups. In triage, where decisions affect prioritization and waiting time, concerns about fairness, representation, and unequal model performance are especially important. Explainability may therefore serve not only as a tool for user acceptance, but also as a mechanism for ethical accountability and critical review of AI-supported decisions^19^.

The present study examined nurses’ perceptions of a specific AI-augmented triage system that functioned as a decision-support tool rather than an autonomous decision-maker. During the study period, the system did not update its decision rules autonomously at the point of care, and nurses retained final authority over triage decisions. This distinction is important because adoption dynamics may differ substantially between static decision-support systems and more adaptive or agentic AI systems. As healthcare AI becomes more autonomous and dynamically embedded in clinical workflows, perceived risk, trust, and accountability concerns may become more complex^42^.

Finally, AI-augmented triage systems should not be viewed as isolated technical tools. Emergency triage decisions are shaped by patient characteristics, resource availability, social context, and environmental pressures. Broader discussions of human-centered and hyper-personalized healthcare emphasize that AI implementation must consider the wider health ecosystem rather than focusing only on algorithmic outputs^43^. Future studies should therefore examine how patient-level heterogeneity, resource constraints, and local implementation contexts influence nurses’ trust, perceived risk, and use of AI-supported triage.

### Practical implications for nursing management

For nursing management, the findings suggest three practical areas for attention. First, nurse leaders may need to involve frontline triage nurses in AI implementation, training, and feedback processes. Because task-technology fit was positively associated with attitude, nurses’ feedback can help identify whether the system supports or disrupts real triage workflow.

Second, training should address not only how to operate the AI system, but also how to interpret and critically evaluate AI-generated recommendations. Training programs may include examples of human—AI agreement and disagreement, appropriate override procedures, documentation expectations, and escalation pathways when AI outputs conflict with clinical judgement.

Third, managers may need to create structured channels for reporting AI-related concerns. Rather than treating discrepancies as individual errors, departments may benefit from reviewing them as opportunities for system learning and quality improvement. This approach is consistent with a just-culture orientation^38,39^, but it should be adapted to local legal, institutional, and clinical governance frameworks. Importantly, these recommendations should be interpreted as implications suggested by the observed associations, not as interventions proven effective by the present design.

## Limitations and future research

Several limitations should be acknowledged. First, this study used a cross-sectional design, which limits causal interpretation. Although the proposed model was informed by established technology adoption theories and implementation literature, the observed relationships should be interpreted as associations rather than evidence of causal effects. Future longitudinal studies are needed to examine how nurses’ attitudes, perceived risk, psychological safety, and actual use of AI-augmented triage systems evolve over time.

Second, all variables were measured using self-reported questionnaires collected from the same respondents at a single time point. Although procedural remedies were used and statistical checks, including Harman’s single-factor test and collinearity diagnostics, suggested that no single factor dominated the data, common method bias cannot be completely ruled out. Future studies should combine survey data with objective indicators, such as system log data, frequency of AI recommendation use, override rates, and documented triage outcomes.

Third, the study was conducted in nine pilot hospitals in Shanghai with relatively mature digital infrastructure and experience using the same AI-augmented triage system. Therefore, the findings may not be generalizable to resource-limited hospitals, rural emergency departments, hospitals using different triage protocols, or healthcare systems with different legal and regulatory environments. Future research should examine whether the same patterns are observed across more diverse institutional, regional, and international settings.

Fourth, the study focused on nurses’ perceptions rather than the technical performance or clinical effectiveness of the AI-augmented triage system. We did not include patient-level data, algorithmic accuracy, calibration, false-positive or false-negative rates, or detailed records of human–AI disagreement. Therefore, the findings should not be interpreted as evidence that the AI system improves triage accuracy or patient outcomes. Future research should integrate perception-based adoption models with objective performance evaluation and patient safety indicators.

Fifth, although the intraclass correlation coefficients suggested limited hospital-level clustering, nurses were still nested within nine hospitals. Shared organizational culture, leadership style, training arrangements, and implementation maturity may have influenced nurses’ perceptions. Because the number of hospital clusters was small, formal multilevel modelling was not conducted. Future studies involving a larger number of hospitals should use multilevel or mixed-effects models to distinguish individual-level adoption factors from hospital-level implementation conditions.

Sixth, although supplementary demographic checks were conducted for gender, age group, and years of emergency experience, the present survey did not directly measure prior AI-related training or hospital-level implementation maturity. These contextual factors may influence nurses’ perceptions of task-technology fit, explainability, psychological safety, perceived risk, and intention to use. Future research should include more detailed measures of training exposure, implementation maturity, and organizational readiness to better examine their potential influence on AI-augmented triage adoption.

Seventh, the study examined intention to use rather than long-term sustained use. Although all included participants had at least three months of actual experience with the AI-augmented triage system, their responses may reflect early post-implementation perceptions rather than stable, routinized use. Future studies should investigate how adoption changes after longer exposure and whether intention translates into consistent use in daily triage practice.

Finally, the study examined a specific AI-augmented triage system that functioned as a decision-support tool rather than an autonomous or adaptive agent. As AI systems become more dynamic, adaptive, and agentic, nurses’ perceived risk, trust, psychological safety, and accountability concerns may differ from those observed in this study. Future research should compare adoption dynamics across systems with different levels of autonomy, explainability, and human oversight.

## Conclusion

This multi-hospital cross-sectional study found that emergency nurses’ intention to use AI-augmented triage systems was associated with both technology-related and human-organizational perceptions. Task-technology fit and perceived explainability were positively associated with nurses’ attitudes toward using AI-augmented triage, while psychological safety also showed a strong positive association with attitude. These findings suggest that emergency nurses’ acceptance of AI-supported triage may depend not only on whether the system fits clinical tasks and provides understandable recommendations, but also on whether nurses feel safe to use, question, or report concerns about AI-generated outputs.

Perceived risk showed a small negative moderating effect on the association between attitude and intention to use. Although this finding should be interpreted cautiously, it suggests that favourable attitudes toward AI-augmented triage may not fully translate into intention to use when nurses perceive clinical, legal, or professional uncertainty. Therefore, AI-augmented triage implementation should address not only system functionality and explainability, but also accountability boundaries, psychological safety, and human–AI collaboration.

Given the cross-sectional and self-reported design, the findings should be interpreted as associations rather than causal evidence. Future longitudinal, multilevel, and mixed-methods studies using objective system-use data and patient safety indicators are needed to further examine how nurses’ perceptions evolve and how AI-augmented triage can be responsibly integrated into emergency care.

## Data Availability

The datasets generated and/or analyzed during the current study are not publicly available due to privacy restrictions but are available from the corresponding author on reasonable request.

## Appendices

### Appendix A: Measurement questionnaire

**Table:**

Variables	Item statement
Task-technology fit (TTF)	The AI-augmented triage system fits well with my daily triage workflow
	The functions provided by the AI system match the specific requirements of my clinical tasks
	The AI system helps me complete triage assessments more efficiently
Perceived explainability (PE)	The AI system provides clear reasons for its triage recommendations
	It is easy for me to understand how the AI system arrives at a specific triage level
	The logic behind the AI’s decision-making process is transparent to me
Psychological safety (PS)	If I make a mistake while using the new AI system, it is not held against me
	I feel safe taking risks or experimenting with the AI system in this department
	My nurse manager supports learning from errors related to AI usage rather than blaming individuals
Perceived risk (PR)	I am concerned that using the AI system may lead to clinical errors for which I am liable
	I worry about the legal uncertainty and potential accountability issues associated with AI-assisted decisions
	Using the AI system exposes me to unnecessary professional risk in my nursing practice
Attitude towards using (AT)	Using the AI-augmented triage system is a good idea
	I have a positive attitude toward using the AI system in my work
	I believe that using the AI system is beneficial for my clinical practice
Intention to use (IU)	I intend to use the AI-augmented triage system in my daily work routine
	I predict I will use the AI system frequently for patient triage
	I plan to continue using the AI system in the future
